# Impact of network treatment in patients with resected pancreatic cancer on use and timing of chemotherapy and survival

**DOI:** 10.1093/bjsopen/zrad006

**Published:** 2023-05-08

**Authors:** Jana S Hopstaken, Pauline A J Vissers, Rutger Quispel, Judith de Vos-Geelen, Lodewijk A A Brosens, Ignace H J T de Hingh, Lydia G van der Geest, Marc G Besselink, Kees J H M van Laarhoven, Martijn W J Stommel

**Affiliations:** Department of Surgery, Radboud University Medical Center, Nijmegen, The Netherlands; Radboud Institute for Health Sciences, Radboud University Medical Center, Nijmegen, The Netherlands; Department of Surgery, Radboud University Medical Center, Nijmegen, The Netherlands; Department of Research and Development, Netherlands Comprehensive Cancer Organisation (IKNL), Utrecht, The Netherlands; Department of Gastroenterology and Hepatology, Reinier de Graaf Groep, Delft, The Netherlands; Department of Internal Medicine, Division of Medical Oncology, Maastricht University Medical Center, GROW, Maastricht University, Maastricht, The Netherlands; Department of Pathology, Radboud University Medical Center, Nijmegen, The Netherlands; Department of Pathology, UMC Utrecht, Utrecht, The Netherlands; Department of Surgery, Catharina Hospital, Eindhoven, The Netherlands; Department of Research and Development, Netherlands Comprehensive Cancer Organisation (IKNL), Utrecht, The Netherlands; Department of Surgery, Amsterdam UMC, University of Amsterdam, Amsterdam, The Netherlands; Cancer Center Amsterdam, Amsterdam UMC, Amsterdam, The Netherlands; Department of Surgery, Radboud University Medical Center, Nijmegen, The Netherlands; Department of Surgery, Radboud University Medical Center, Nijmegen, The Netherlands

## Abstract

**Background:**

Centralization of pancreatic cancer surgery aims to improve postoperative outcomes. Consequently, patients with pancreatic cancer may undergo pancreatic surgery in an expert centre and adjuvant chemotherapy in a local hospital (network treatment). The aim of this study was to assess whether network treatment has an impact on time to chemotherapy, failure to complete adjuvant chemotherapy, and survival. Second, whether these parameters varied between pancreatic networks was studied.

**Methods:**

This retrospective study included all patients diagnosed with non-metastatic pancreatic ductal adenocarcinoma who underwent pancreatic surgery and adjuvant chemotherapy, registered in the Netherlands Cancer Registry (2015–2020). Time to chemotherapy was defined as the time between surgery and the start of adjuvant chemotherapy. Completion of adjuvant chemotherapy was defined as the receipt of 12 cycles of FOLFIRINOX or six cycles of gemcitabine. Analysis was performed with linear mixed models and multilevel logistic regression models. Cox regression analyses were performed for survival.

**Results:**

In total, 1074 patients were included. Network treatment was observed in 468 patients (43.6 per cent) and was not associated with longer time to chemotherapy (0.77 days, standard error (s.e.) 1.14, *P* = 0.501), failure to complete adjuvant chemotherapy (odds ratio (OR) = 1.140, 95 per cent c.i. 0.86 to 1.52, *P* = 0.349), and overall survival (hazards ratio (HR) = 1.04, 95 per cent c.i. 0.88 to 1.22, *P* = 0.640). Significant variation between the networks was observed for time to chemotherapy (range 40.5–63 days, *P* < 0.0001) and completion of adjuvant chemotherapy (range 19–52 per cent, *P* = 0.030). Adjusted for case mix, time to chemotherapy significantly differed between networks.

**Conclusion:**

In this nationwide analysis, network treatment in patients with resected pancreatic cancer was not associated with longer time to chemotherapy, failure to complete adjuvant chemotherapy, and worse survival. Significant variation between pancreatic cancer networks was found for time to chemotherapy.

## Teaser

This study shows that 44 per cent of all patients with pancreatic cancer treated with surgery and adjuvant chemotherapy undergo these in two hospitals (network treatment) instead of one pancreatic centre. It was hypothesized that network treatment, in which a patient is transferred from a referring hospital to a pancreatic centre, could be associated with the use and timing of adjuvant chemotherapy. In this study we have shown that network treatment does not negatively impact these outcomes.

## Introduction

Over the past decade, many European countries have centralized surgery of the oesophagus, liver, rectum, and pancreas^1–3^. The main reasons for these centralization policies were improved patient outcomes and cost savings^1^. For pancreatic surgery, studies demonstrated that centralization was associated with lower in-hospital mortality rates and improved long-term survival, prompting policymakers to centralize pancreatic surgery in the Netherlands in 2011, with a minimum case volume per hospital of 20 pancreatoduodenectomies per year^4–7^. Since then, two types of hospitals can be distinguished. Pancreatic centres have a specialized multidisciplinary team (MDT) and perform pancreatic surgery, whereas referring hospitals, or non-pancreatic centres, do not perform pancreatic surgery but provide other parts of pancreatic cancer care (such as diagnostics, chemotherapy, or best supportive care). Together, these centres form pancreatic cancer networks. Cancer networks are required to meet quality standards as formulated by the Dutch Federation of Oncologic Societies (SONCOS)^8^.

Patients with pancreatic cancer eligible for curative treatment require a multimodality treatment consisting of pancreatic surgery and (neo)adjuvant chemotherapy^9,10^. In pancreatic cancer networks, patients may undergo surgery in the pancreatic centre and receive adjuvant chemotherapy in a non-pancreatic centre. This is network treatment or multicentre treatment. Because non-pancreatic centres are more abundant and patients may prefer to receive chemotherapy closer to home, the expectation is that multicentre treatment is common. However, the extent of multicentre treatment in pancreatic cancer patients and whether this has an impact on time to chemotherapy, failure to complete chemotherapy, and survival, is unknown. Multicentre treatment requires a transfer of patient information from one centre to another, with different customs of care, and a change of the primary treating physician. This is logistically demanding and could possibly result in delay or failure to complete chemotherapy. It is relevant to assess whether multicentre care is associated with these outcomes, as this could have implications for clinical practice. Limited studies have focused on multicentre treatment in oncological patients, and those that are available were mainly descriptive or included only a low sample size of patients with pancreatic cancer^11,12^.

The aim of this study was to assess the nationwide extent of multicentre treatment in patients diagnosed with pancreatic cancer and whether this was associated with time to chemotherapy, failure to complete chemotherapy, and survival. Second, the variation between pancreatic cancer networks for these outcomes was assessed.

## Methods

### Ethical consideration

According to the Dutch Medical Research Involving Human Subjects Act (WMO) and the Central Committee on Research Involving Human Subjects, no ethical approval for this study was required, as this study used data from the Netherlands Cancer Registry (NCR). The privacy board of the NCR as well as the scientific committee of the Dutch Pancreatic Cancer Group (DPCG) approved the study protocol^13^.

### Study design

This was a population-based, nationwide, retrospective study of patients registered in the NCR, containing data on all newly diagnosed cancer patients in the Netherlands, populated by approximately 17 million inhabitants. These diagnoses are notified to the NCR by the automated pathological archive (PALGA). Specially trained data managers retrieve data concerning treatment, and patient and tumour characteristics from medical records. Through annual linkage of the NCR with the Municipal Personal Records database, data on vital status were available, with follow-up completed until 1 February 2021.

### Study population and data collection

All patients with non-metastatic (on imaging) pancreatic ductal adenocarcinoma (PDAC) or suspected PDAC (ICD-O C2, morphology codes 8010, 8012, 8020, 8021, 8035, 8070, 8140, 8144, 8154, 8163, 8211, 8310, 8480, 8490, 8500, and 8560) diagnosed between 2015 and 2020 were included. Patients were excluded from analysis if they did not receive pancreatic surgery and adjuvant chemotherapy or in the case of an age less than 18 years, diagnosis and treatment abroad, or diagnosis by incidental finding during treatment for other types of tumours (such as surgery for kidney cancer leading to diagnosis of PDAC). Information on patient characteristics (such as age, sex, ASA score, and co-morbidity), tumour characteristics (such as morphology, location in the pancreas, differentiation grade, and tumour diameter according to pathology report), and treatment and care-related characteristics (such as preoperative biliary drainage, type of treatment (for example neoadjuvant chemo(radio)therapy, type of pancreatic resection, and adjuvant chemotherapy), surgical margin status, and length of hospital stay (LOS)) was available. In addition, information on time of first visit, time of diagnosis, time of first tumour treatment, time between surgery and adjuvant chemotherapy, and treatment location, that is pancreatic centre or non-pancreatic centre, was available.

### Definitions

Multicentre treatment was defined as patients receiving surgery in a pancreatic centre and adjuvant chemotherapy in a non-pancreatic centre, that is referring hospitals. Monocentre treatment included patients who had these two treatment modalities in one pancreatic centre. Time to chemotherapy was defined as the interval between the day of surgery and the start of adjuvant chemotherapy (days). The National Comprehensive Cancer Network (NCCN) guideline and the Dutch national guideline for pancreatic cancer recommend to start adjuvant chemotherapy within 12 weeks after surgery^9,10^. To study associations with time to chemotherapy, a prolonged LOS was expected to be an important covariable and a proxy for postoperative complications. A prolonged LOS was defined as a duration exceeding the 75th percentile^14^, which was greater than 16 days in this cohort. Completion of adjuvant chemotherapy was defined as the completion of all cycles of chemotherapy, that is 12 cycles for adjuvant (modified) FOLFIRINOX chemotherapy and six cycles for gemcitabine-based chemotherapy. For patients who received neoadjuvant chemo(radio)therapy, the sum of both neoadjuvant and adjuvant regimens was considered. In the case of patients having received different regimens as neoadjuvant and adjuvant treatment, a total duration of at least 24 weeks was perceived as completion of chemotherapy. Patients who received less than these cut-offs, failed to complete chemotherapy.

### Statistical analysis

Descriptive statistics are expressed as mean and standard deviation (s.d.) or as median and interquartile range (i.q.r.) for continuous variables. Categorical variables are expressed as numbers and percentages. Differences between monocentre and multicentre treatment were assessed using chi-squared tests or Mann–Whitney *U* tests. Network differences were assessed using Fisher’s exact test and the Kruskal–Wallis test. The association between multicentre treatment and time to chemotherapy was assessed using Generalized Linear Mixed Models (GLMM). For the association with failure to complete chemotherapy, multivariable multilevel logistic analysis was performed. The variation in outcome explained by network was determined using the intraclass correlation coefficient (ICC)^15^. Estimates (beta) and standard error (s.e.) or odds ratio (OR) with a 95 per cent c.i. are reported. Variables for adjustment were selected based on statistical significance in the univariable logistic regression analysis (*P* < 0.10), clinical relevance, or if they were deemed a confounder. Survival distributions were analysed by means of Kaplan–Meier curves and compared using a log rank test. Survival time was defined as the time between adjuvant chemotherapy initiation and the date of death or censoring. A multilevel Cox regression analysis was performed to study associations between multicentre treatment and survival, presented as hazards ratios (HRs). Two-sided *P* values of <0.05 were considered statistically significant. Statistical analyses were performed using SPSS^®^ version 25 (IBM, Armonk, NY, USA), SAS^®^ version 9.4 (SAS Institute, Cary, NC, USA), and R studio version 1.4.1103.

## Results

### Baseline characteristics

**Fig. 1:**
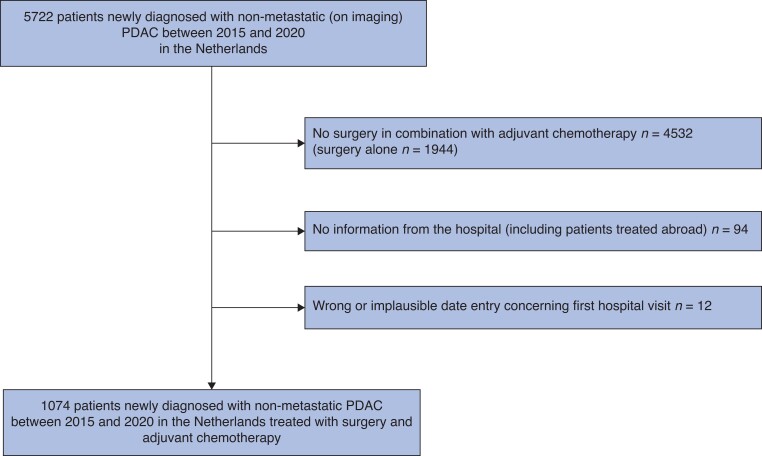
Patient selection from the Netherlands Cancer Registry PDAC, pancreatic ductal adenocarcinoma.

In total, 5722 patients with non-metastatic PDAC diagnosed between 2015 and 2020 were identified in the registry. Of these patients, 4532 (79 per cent) did not undergo pancreatic surgery in combination with adjuvant chemotherapy. Of 94 patients (2 per cent) no information from the hospital was available and 12 patients (0.2 per cent) had implausible data and were excluded from analysis (*Fig. 1*). Therefore, 1074 patients were included in the final analysis.

**Fig. 2:**
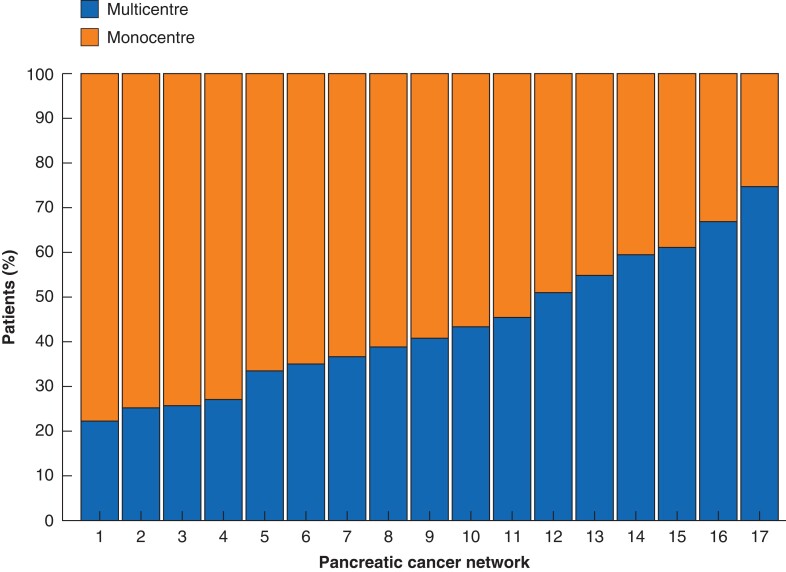
Percentage of patients with pancreatic ductal adenocarcinoma undergoing multicentre or monocentre treatment per pancreatic cancer network

Monocentre treatment was performed in 606 patients (56.4 per cent) and multicentre treatment in 468 patients (43.6 per cent). Patients undergoing monocentre treatment significantly more often received neoadjuvant chemotherapy (24.1 per cent *versus* 6.4 per cent, *P* < 0.001). There were no statistically significant differences in the administered type of adjuvant chemotherapy (for example FOLFIRINOX or gemcitabine-based therapy) between patients undergoing monocentre and multicentre treatment (*P* = 0.175) (*Table 1*). Seventeen pancreatic cancer networks were identified. The percentage of patients undergoing multicentre treatment significantly differed between the networks, ranging from 22 to 75 per cent (*P* < 0.001) (*Fig. 2*).

**Table 1:** Baseline characteristics of 1074 patients with pancreatic ductal adenocarcinoma undergoing monocentre or multicentre treatment

	Monocentre (*n* = 606)	Multicentre* (*n* = 468)	*P*
**Patient characteristics**			
Age (years), median (i.q.r.)	66 (58–72)	67 (60–72)	0.018†
Sex			
Male	326 (53.8)	256 (54.7)	
Female	280 (46.2)	212 (45.3)	0.768
ASA classification			0.713
I	57 (9.4)	49 (10.5)	
II	376 (62)	286 (61.1)	
III–IV	135 (22.3)	110 (23.5)	
Missing	38 (6.3)	23 (4.9)	
Charlson co-morbidity index ≥2	68 (12.7)	61 (13.9)	0.741
Missing	69 (11.4)	29 (6.2)	
Tumour location, pancreatic head	474 (78.2)	376 (80.5)	0.693
Tumour diameter during pathology assessment (cm), median (i.q.r.)	3 (2.3–4.0)	3.0 (2.4–4.0)	0.576
Tumour diameter categories			0.202
0–2 cm	100 (16.8)	95 (20.5)	
>2–4 cm	366 (61.4)	262 (56.5)	
>4 cm	130 (21.8)	107 (23.1)	
**Treatment-related characteristics**			
MDT meeting performed	556 (92.6)	447 (95.5)	0.042†
Biliary drainage (preoperative)	314 (51.8)	228 (48.7)	0.314
Neoadjuvant chemotherapy	146 (24.1)	30 (6.4)	<0.001†
Type of surgery			0.061
Pancreatoduodenectomy	270 (44.6)	178 (38)	
PPPD	218 (36)	200 (42.7)	
PRPD	15 (2.5)	14 (3.0)	
Body/tail resection	92 (15.2)	72 (15.4)	
Total pancreatectomy	10 (1.7)	2 (0.4)	
Other	1 (0.2)	2 (0.4)	
Radicality of resection			0.295
R0	334 (55.1)	265 (56.6)	
R1	266 (43.9)	202 (43.2)	
Unknown/not determined	6 (1)	1 (0.2)	
Prolonged LOS	84 (14.8)	69 (15.4)	0.792
Time to chemotherapy (days), median (i.q.r.)	56 (44–69)	55 (44–68)	0.900
Type of adjuvant chemotherapy			0.175
FOLFIRINOX regimen	96 (15.8)	90 (19.2)	
Gemcitabine-based regimen	501 (82.7)	367 (78.4)	
Other	9 (1.5)	11 (2.4)	
Failure to complete adjuvant chemotherapy	203 (34.5)	174 (37.9)	0.258
Failure to complete adjuvant chemotherapy per regimen			
FOLFIRINOX regimen	51 (54.3)	42 (47.7)	0.379
Gemcitabine regimen	147 (30.2)	130 (35.9)	0.082
Other	5 (62.5)	2 (22.2)	0.153

Values are *n* (%) unless otherwise indicated. *Multicentre treatment is defined as surgery in a pancreatic centre and adjuvant chemotherapy in a non-pancreatic centre. †Values are statistically significant. i.q.r., interquartile range; MDT, multidisciplinary team; PPPD, pylorus-preserving pancreatoduodenectomy; PRPD, pylorus-resecting pancreatoduodenectomy; LOS, length of hospital stay.

The rates of neoadjuvant chemotherapy significantly differed between the networks (median 12.7 per cent, range 0–34.6 per cent, *P* < 0.0001).

### Multicentre treatment and outcomes of interest - time to chemotherapy

Patients with monocentre treatment had a median of 56 (i.q.r. 44–69) days between surgery and initiation of chemotherapy *versus* a median of 55 (i.q.r. 44–68) days for patients undergoing multicentre treatment (*P* = 0.900). Initiation of chemotherapy beyond 12 weeks occurred in 49 (8.1 per cent) patients undergoing monocentre treatment and 51 (10.9 per cent) patients undergoing multicentre treatment (*P* = 0.107). Multicentre treatment was not associated with increased time to chemotherapy in the univariable analysis, with 0.125 days later start of chemotherapy (s.e. 1.13, *P* = 0.912), or in the multivariable analysis (0.768 days, s.e. 1.14, *P* = 0.501). Covariables that were associated with longer time to chemotherapy were age, prolonged LOS, and neoadjuvant chemotherapy (*Table 2*).

**Table 2:** Univariable and multivariable multilevel analysis for the association between multicentre treatment, time to chemotherapy, and failure to complete chemotherapy, including patients with neoadjuvant chemotherapy

	Time to chemotherapy*				Failure to complete chemotherapy			
	Univariable (*n* = 1074)		Multivariable (*n* = 1005)		Univariable (*n* = 1047)		Multivariable (*n* = 1036)	
	Estimate (s.e.)	*P*	Estimate (s.e.)	*P*	OR (95% c.i.)	*P*	OR (95% c.i.)	*P*
**Fixed effects**								
Age (years)	0.263 (0.0594)‡	<0.0001‡	0.193 (0.0606)‡	0.002‡	1.01 (1.00,1.025)	0.145		
ASA								
I	−1.886 (1.849)	0.308	−1.434 (1.818)	0.430	0.76 (0.47,1.235)	0.254	0.758 (0.471,1.22)	0.247
II	Ref		Ref		Ref		Ref	
III–IV	3.225 (1.324)‡	0.015‡	1.449 (1.327)	0.275	1.59 (1.16,2.17)‡	0.005‡	1.524 (1.111,2.089)‡	0.010‡
Missing	−1.471 (2.495)	0.556	−1.445 (2.456)	0.556	1.25 (0.67,2.34)	0.484	1.274 (0.679,2.39)	0.442
Preoperative biliary drainage								
No	Ref				Ref			
Yes	1.204 (1.091)	0.27			0.840 (0.64,1.11)	0.195		
Neoadjuvant chemotherapy								
No	Ref		Ref		Ref			
Yes	4.11 (1.51)‡	0.0065‡	4.406 (1.619)‡	0.007‡	0.72 (0.48,1.08)	0.105		
Tumour diameter (pathology)								
0–2 cm	Ref				Ref			
>2–4 cm	−1.904 (1.458)	0.1921			1.22 (0.84,1.77)	0.279		
>4 cm	−2.787 (1.809)	0.1236			1.42 (0.91,2.22)	0.119		
Radical resection								
R0	Ref				Ref			
R1	−1.904 (1.458)	0.183			1.20 (0.918,1.58)	0.191		
Not able to determine	−2.787 (1.809)	0.725			0.400 (0.04,3.97)	0.414		
Number of PLN	−0.288 (0.161)	0.0747	−0.196 (0.161)	0.224	1.05 (1.01,1.09)‡	0.008‡	1.051 (1.013,1.091)‡	0.009‡
Prolonged LOS†								
No	Ref		Ref		Ref			
Yes	15.494 (1.497)‡	<0.0001‡	14.867 (1.494)‡	<0.0001‡	1.16 (0.78,1.71)	0.442		
Multicentre treatment								
No	Ref		Ref		Ref		Ref	
Yes	0.124 (1.132)	0.912	0.768 (1.14)	0.501	1.15 (0.87,1.53)	0.315	1.14 (0.86,1.52)	0.349
**Random effects§**								
ICC	NA		5.87	0.015‡	NA		1.06	0.199
Range of clusters								
Lowest	NA		−6.447 (2.418)	0.008‡	NA		0.853 (0.592,1.229)	0.394
Highest	NA		8.972 (1.8296)	<0.0001‡	NA		1.217 (0.8,1.851)	0.359

In the univariable analysis, only parameters with *P* < 0.10, except for the central determinant, were considered in the multivariable analysis. *Number of days, calculated with generalized linear mixed models. †Defined as greater than the 75th percentile of LOS (i.e. >16 days). ‡Values are statistically significant. §In the multilevel model, only a random intercept was applied because random slopes were not required. s.e., standard error; PLN, positive lymph nodes; LOS, length of hospital stay; ICC, intraclass correlation coefficient; NA, not applicable.

**Fig. 3:**
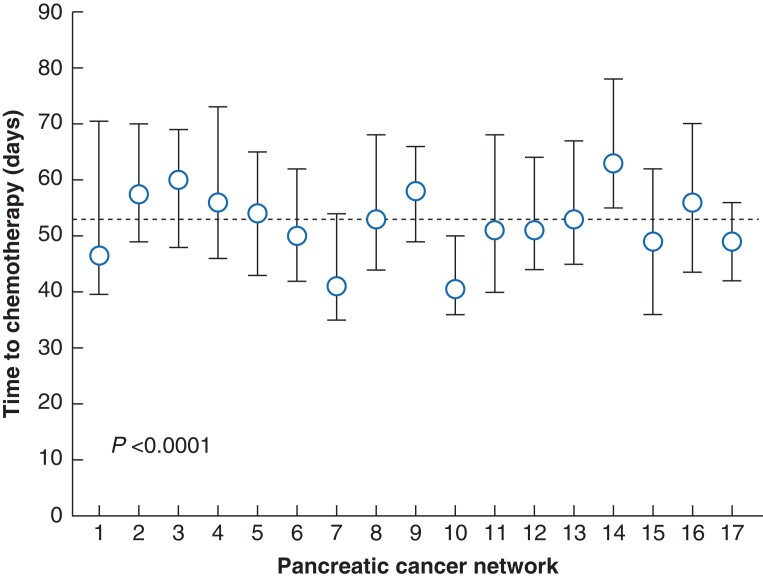
Time to chemotherapy for patients with pancreatic ductal adenocarcinoma per pancreatic cancer network, represented as median (circle) and interquartile range (whiskers); the dotted line at 53 days is the median number of days in total

Significant variation in time to chemotherapy, regardless of case mix, was present between the pancreatic cancer networks, with a range of medians of 40.5 to 63 days (*P* < 0.0001) (*Fig. 3*.)

In the multilevel multivariable analysis, the ICC was 6 per cent (*P* = 0.0148). This indicates that 6 per cent of the total variation in time to chemotherapy could be attributed to the network. Corrected for case mix, one network had significantly less time to chemotherapy (6.4 days (s.e. 2.42), *P* = 0.0078) and one network had significantly longer time to chemotherapy (9.0 days (s.e. 1.83), *P* < 0.0001) compared with the network median.

### Multicentre treatment and outcomes of interest - failure to complete adjuvant chemotherapy

There were no statistically significant differences in the administered type of adjuvant chemotherapy between monocentre and multicentre patients (*P* = 0.175) (*Table 1*). A FOLFIRINOX-based regimen was provided to 16 per cent and 19 per cent of monocentre and multicentre patients respectively, whereas a gemcitabine-based regimen was provided to 83 per cent and 79 per cent of monocentre and multicentre patients respectively. Failure to complete adjuvant chemotherapy was observed in 35 per cent of patients with monocentre treatment *versus* 38 per cent of patients undergoing multicentre treatment (*P* = 0.258). In multilevel logistic regression analysis, no association was observed between multicentre treatment and failure to complete adjuvant chemotherapy (OR 1.14, 95 per cent c.i. 0.856 to 1.515, *P* = 0.349). Covariables that were associated with failure to complete chemotherapy were ASA score III–IV (OR 1.52, 95 per cent c.i. 1.11 to 2.09, *P* = 0.010) and positive lymph nodes (OR 1.05, 95 per cent c.i. 1.01 to 1.09, *P* = 0.009) (*Table 2*).

**Fig. 4:**
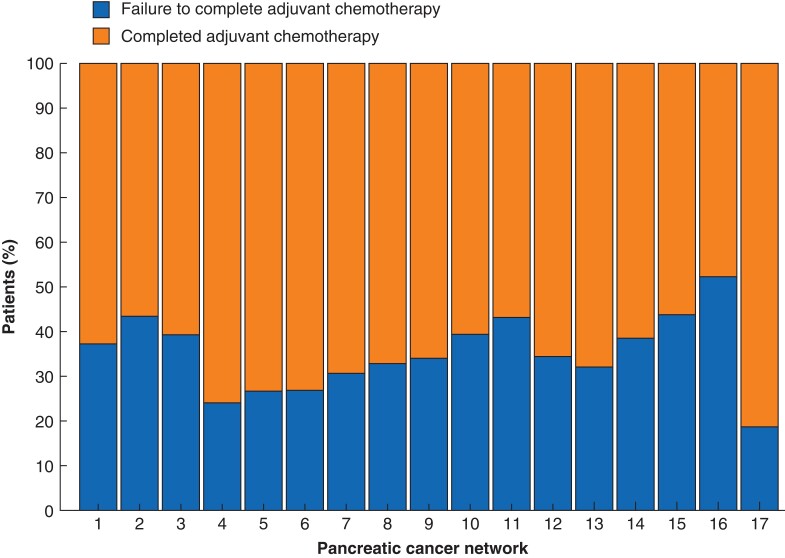
Percentage of failure to complete adjuvant chemotherapy in patients with pancreatic ductal adenocarcinoma per pancreatic cancer network

Significant variation in failure to complete adjuvant chemotherapy, regardless of case mix, was observed between networks, ranging from 19 per cent to 52 per cent (*P* = 0.030) (*Fig. 4*). In multilevel analysis, with correction of ASA score, positive lymph nodes, and multicentre treatment, there were no significant differences between the networks in ORs for failure to complete adjuvant chemotherapy. The variation attributed to the network was minimal and not statistically significant (ICC 1.06 per cent, *P* = 0.199).

The effect of multicentre treatment on time to chemotherapy and failure to complete chemotherapy was also assessed for the study population excluding patients with neoadjuvant chemotherapy and yielded similar results (*Table S1*).

### Survival

The median overall survival for patients undergoing multicentre treatment was 23.4 (i.q.r. 12.6–44.4) months compared with 25.7 (i.q.r. 12.9–52.8) months for monocentre treatment (*P* = 0.224). In multilevel Cox regression analysis, multicentre treatment was not associated with overall survival (HR 1.04, 95 per cent c.i. 0.88 to 1.22, *P* = 0.64). Covariables that were associated with worse survival were ASA I, tumour diameter greater than 2 cm, and R1 resection margin status (*Table 3*). Failure to complete adjuvant chemotherapy was, in contrast to time to chemotherapy, significantly associated with survival (HR 1.30, 95 per cent c.i. 1.10 to 1.531, *P* = 0.002).

**Table 3:** Multilevel multivariable Cox regression analysis of overall survival for all patients with resection and adjuvant chemotherapy

	HR (95% c.i.)	*P*
**Fixed effects**		
Age	1.00 (0.66,1.01)	0.880
Sex	1.06 (0.90,1.24)	0.510
ASA		
I	1.43 (1.11,1.84)*	0.0054*
II	Ref	
III—IV	1.00 (0.82,1.22)	0.980
Missing	1.14 (0.79,1.65)	0.490
Tumour diameter (pathology) (cm)		
0–2	Ref	
>2–4	1.28 (1.01,1.63)*	0.041*
>4	1.98 (1.51,2.60)*	<0.0001*
Radical resection		
R0	Ref	
R1	1.44 (1.23,1.70)*	<0.0001*
Not able to determine	0.27 (0.04,1.96)	0.210
Neoadjuvant chemotherapy		
No	Ref	
Yes	0.85 (0.65,1.10)	0.210
Time to chemotherapy	0.999 (0.995,1.00)	0.820
Failure to complete chemotherapy		
No	Ref	
Yes	1.300 (1.10,1.53)*	0.0018*
Multicentre treatment		
No	Ref	
Yes	1.04 (0.88,1.22)	0.640
**Random effects**		
Pancreatic cancer networks	NA	0.610

*Values are statistically significant. HR, hazards ratio; NA, not applicable.

## Discussion

This nationwide study shows that approximately half of all patients with PDAC with a curative treatment undergo multicentre treatment, that is surgery in a pancreatic centre followed by adjuvant chemotherapy in a non-pancreatic centre. Despite potential logistical hurdles, multicentre treatment was not associated with longer time to chemotherapy, failure to complete chemotherapy, or worse overall survival compared with monocentre treatment. Significant variation between pancreatic networks was present for time to chemotherapy and for failure to complete chemotherapy. Adjusted for case mix, this variation remained for time to chemotherapy. Six per cent of the variation in time to chemotherapy could be attributed to the variation between networks.

Multicentre treatment in patients with pancreatic cancer has previously been described in two registry-based studies from the USA. One study by Clarke *et al*.^11^ in 2017, including 32 patients with pancreatic cancer, described multicentre treatment in 47 per cent of patients, which is similar to our findings (44 per cent). They did not study outcomes of multicentre treatment. The other study, by Shannon *et al*.^12^ in 2020, described survival outcomes for 380 patients with pancreatic cancer after single-centre or multicentre treatment. They also did not report significant differences in overall survival for monocentre and multicentre patients^12^. However, the proportion of patients undergoing multicentre treatment (20 per cent) was considerably lower compared with the 44 per cent in our study^12^. With only 1 per cent of the study population being female, the patient population was different, and the results less generalizable, compared with our study. Both US studies did not study the impact of multicentre treatment other than the impact on survival^11,12^.

In this analysis, failure to complete chemotherapy had a significant impact on survival, whereas time to chemotherapy did not. These results thereby confirm what was previously described in the ESPAC-3 trial. This long-term survival study observed that completion of all cycles rather than early initiation of chemotherapy was an important independent favourable prognostic factor for survival^16,17^. Therefore, the authors concluded that optimal postoperative recovery and subsequent completion of all cycles is more important.

This study has limitations. First, it used retrospective, registry-based data. These data permit imbalances of prognostic factors between the two studied groups. Important covariables for completion of chemotherapy, which we could not account for, were recurrence of disease, response to chemotherapy, and toxicity. Second, this study was performed in the Dutch healthcare setting with relatively small distances between hospitals. This should be taken into account when extrapolating these findings to other healthcare settings. A final limitation is that no data concerning the treatment advice provided by the MDT were available. Thus, we could not identify patients for whom treatment advice was constructed by the MDT that included adjuvant chemotherapy but did not eventually receive it. Because this information was lacking, the authors could not classify these patients in monocentre or multicentre treatment and could not study the effect of network treatment on the omission of chemotherapy.

A strength of this study is that it is a large population-based study in which network treatment in patients with PDAC and its associations with quality indicators and survival are studied. This is meaningful as previous studies have shown that organization of healthcare has an impact on patient outcomes, as illustrated by studies on centralization of pancreatic surgery^1,4–7^. As research on pancreatic healthcare services is scarce^18^, this study provides additional knowledge on possible effects of organization of healthcare for pancreatic cancer patients. A second strength is that the authors investigated how much of the variation in the outcomes could be attributed to the pancreatic cancer network by means of multilevel analysis. This is methodologically more appropriate than ordinary least squares regression analysis and provides insight into the variation attributed to the network^15^.

Future oncological healthcare services will most probably further centralize complex oncological care. For pancreatic surgery in the Netherlands, a decrease in hospitals performing pancreatic surgery was observed from 56 centres in 2004 to 15 centres in 2022^4,19^. It is conceivable that the number of centres will further decrease to meet quality criteria and cost-effectiveness. Subsequent increased fragmented care as a result of multicentre treatment and increasing travel distances for patients could have negative effects. A Dutch study assessing the changes in travel distances as a result of centralization of surgery over the interval of 2006–2017 for patients with pancreatic, oesophageal, and gastric cancer reported increased travel distances and travel burden^20^. One-third of patients indicated they preferred surgery closer to home than the expert centre. In particular, patients living in remote areas, patients with a lower socio-economic status, and the elderly are reported to experience increased travel burden in large centralized networks^20–23^. These aspects should be adequately considered in policymaking to avoid increasing healthcare disparities, especially as healthcare policymaking is predominantly considered from a professional and organizational perspective and to a lesser extent from a patient perspective^24^.

For the time being, this study supports the current organization of pancreatic network care with multicentre treatment as it does not affect timing and completion of chemotherapy or survival. Network variation in these outcomes was present, especially for time to chemotherapy. Based on this study, the authors cannot exactly pinpoint which elements could explain this variation. Other studies are required to study the underlying reasons for the variation between pancreatic cancer networks.

In conclusion, in patients with resected pancreatic cancer, multicentre treatment was not associated with time to chemotherapy, completion of chemotherapy, and survival compared with patients undergoing monocentre treatment. Significant variation between pancreatic cancer networks was found for time to chemotherapy. This should be addressed in future studies.

## Supplementary Material

zrad006_Supplementary_Data

## Data Availability

Data from this study can be made available upon reasonable request via the Netherlands Cancer Registry or by the corresponding author.
