# Supplementary material for: Impact of network treatment in patients with resected pancreatic cancer on use and timing of chemotherapy and survival
Source: BJS Open. 2023 May 8;7(3):zrad006. doi: 10.1093/bjsopen/zrad006 (PMC10165062; doi:10.1093/bjsopen/zrad006)
Supplement: zrad006_Supplementary_Data [file zrad006_supplementary_data.docx]

**Impact of network treatment in patients with resected pancreatic cancer on use and timing of chemotherapy and survival**

Jana S. Hopstaken^1,2^, Pauline A.J. Vissers^1,3^, Rutger Quispel^4^, Judith de Vos-Geelen^5^, Lodewijk A.A. Brosens^6^, Ignace de Hingh^7^, Lydia G. van der Geest^3^, Marc G. Besselink^8,9^, Kees J.H.M. van Laarhoven^1^, Martijn W.J. Stommel^1^ for the Dutch Pancreatic Cancer Group

**Affiliations:**
^1^ Department of Surgery, Radboud university medical center, Nijmegen, the Netherlands.
^2^ Radboud Institute for Health Sciences, Radboud university medical center, Nijmegen, the Netherlands.
^3^ Department of Research and Development, Netherlands Comprehensive Cancer Organisation (IKNL), Utrecht, the Netherlands.
^4^ Department of Gastroenterology and Hepatology, Reinier de Graaf Groep, Delft, The Netherlands.
^5^ Department of Internal Medicine, Division of Medical Oncology, Maastricht University Medical Center, GROW, Maastricht University, Maastricht, the Netherlands.
^6^ Department of Pathology, Radboud university medical center, Nijmegen, the Netherlands, Department of Pathology, UMC Utrecht, Utrecht, the Netherlands.
^7^ Department of Surgery, Catharina Hospital, Eindhoven, the Netherlands.
^8^ Department of Surgery, Amsterdam UMC, location University of Amsterdam, Amsterdam, the Netherlands.

^9^ Cancer Center Amsterdam, the Netherlands.

**Corresponding author:**Dr. M.W.J. Stommel, MD, PhD, Hepatopancreatobiliary surgeon
Department of Surgery, Radboud university medical center
Geert Grooteplein 10 (route 618) 6525 GA Nijmegen, The Netherlands.
Tel: +31 024 36 680 86
Mail: [Martijn.Stommel@Radboudumc.nl](mailto:Martijn.Stommel@Radboudumc.nl)
ORCID: [0000-0002-4257-5254](http://orcid.org/0000-0002-4257-5254) 
(during review process: [Jana.Hopstaken@Radboudumc.nl](mailto:Jana.Hopstaken@Radboudumc.nl))

**Supplementary Materials - Index**

| **Supplementary Figures and Tables** |  |
| --- | --- |
| Table S1 | *Pag. 3* |
| **References** | *Pag. 4* |
|  |  |

**Supplementary Figures and Tables**

**Table S1.** *Univariable and multivariable multilevel analysis for the association between multicentre treatment, time-to-chemo and failure to complete chemotherapy, excluding patients with neoadjuvant chemotherapy*

|  | Time-to-chemotherapy | | | | Failure to complete chemotherapy | | | |
| --- | --- | --- | --- | --- | --- | --- | --- | --- |
|  | Univariable  (n = 898) | | Multivariable (n = 852) | | Univariable (n = 886) | | Multivariable*** (n=886) | |
|  | Estimate (SE) | P-value | Estimate (SE) | P-value | OR (95% CI) | P-value | OR (95% CI) | P-value |
| **Fixed effects** |  |  |  |  |  |  |  |  |
| Age | **0.273 (0.0655)** | **<0.001** | **0.190 (0.065)** | **0.0039** | 1.012 (0.997-1.028) | 0.122 |  |  |
| ASA 1 2  3-4  *Missing* | -2.484 (2.010) Ref  2.355 (1.423)  -0.405 (2.895) | 0.216  0.098  0.888 | -2.248 (1.932)  Ref  0.390 (1.402)  0.500 (2.846) | 0.245  0.780  0.860 | 0.689 (0.412-1.154)  Ref  **1.569 (1.124-2.192)**  1.21 (0.551-2.278) | 0.1526  **0.0093**  0.7473 | 0.69 (0.411-1.156)  Ref  **1.504 (1.073-2.107)**  1.116 (0.5490-2.268) | 0.1545  **0.0189**  0.7569 |
| Preoperative biliary drainage  No Yes | Ref 0.961 (1.187) | 0.418 | - | - | Ref  0.916 (0.681-1.232) | 0.5392 | - | - |
| Tumor diameter (pathology)  0-2 cm  >2-4 cm  >4 cm | Ref  -2.26 (1.6)  -2.96 (2.021) | 0.180 0.142 | - | - | Ref  1.081 (0.714-1.635)  1.2 (0.735-1.957) | 0.7044  0.4541 | - | - |
| Radical resection R0  R1  Not able to determine | Ref  **2.256 (1.200)**  -10.595 (7.895) | Ref **0.06** 0.18 | Ref  5.985 (7.467)  8.255 (7.493) | 0.426 0.271 | Ref  1.134 (0.845-1.522)  0.451 (0.043-4.71) | 0.3833  0.4867 | - | - |
| Number of positive lymph nodes | -0.303 (0.171) | 0.078 | **-0.344 (0.169)** | **0.042** | **1.043 (1.003-1.084)** | **0.0349** | **1.043 (1.003-1.085)** | **0.0329** |
| Prolonged hospital LOS No  Yes | Ref  15.377 (1.600) | **<0.0001** | **Ref**  **14.961 (1.598)** | **<0.0001** | Ref  1.132 (0.745-1.718) | 0.5392 | - | - |
| Multicentre treatment  No Yes | Ref 0.704 (1.229) | 0.566 | Ref  0.528 (1.189) | 0.656 | Ref  1.017 (0.751-1.378) | 0.9063 | Ref 1.028 (0.762-1.388) | 0.8461 |
| **Random effects** |  | |  |  |  |  |  |  |
| ICC | NA | | **6.63** | **0.0127** | NA | | *** |  |
| *Range OR of clusters:* |  | |  |  |  | |  |  |
| Lowest | NA | | **-6.447 (2.418)** | **0.0078** | NA | | *** |  |
| Highest | NA | | **8.972 (1.829)** | **<0.0001** | NA | | *** |  |

***A multilevel model was not possible as there was no variation between networks. Therefore multivariable logistic regression analysis was performed.

**References**

1. Vonlanthen R, Lodge P, Barkun JS, et al. Toward a Consensus on Centralization in Surgery. *Annals of Surgery*. 2018;268(5):712-724. doi:10.1097/sla.0000000000002965

2. Stitzenberg KB, Meropol NJ. Trends in centralization of cancer surgery. *Ann Surg Oncol*. Nov 2010;17(11):2824-31. doi:10.1245/s10434-010-1159-0

3. Wouters MWJM, Karim-Kos HE, le Cessie S, et al. Centralization of Esophageal Cancer Surgery: Does It Improve Clinical Outcome? *Annals of Surgical Oncology*. 2009/07/01 2009;16(7):1789-1798. doi:10.1245/s10434-009-0458-9

4. de Wilde RF, Besselink MG, van der Tweel I, et al. Impact of nationwide centralization of pancreaticoduodenectomy on hospital mortality. *Br J Surg*. Mar 2012;99(3):404-10. doi:10.1002/bjs.8664

5. Gooiker GA, van Gijn W, Wouters MW, Post PN, van de Velde CJ, Tollenaar RA. Systematic review and meta-analysis of the volume-outcome relationship in pancreatic surgery. *Br J Surg*. Apr 2011;98(4):485-94. doi:10.1002/bjs.7413

6. Polonski A, Izbicki JR, Uzunoglu FG. Centralization of Pancreatic Surgery in Europe. *J Gastrointest Surg*. Oct 2019;23(10):2081-2092. doi:10.1007/s11605-019-04215-y

7. Latenstein AEJ, Mackay TM, van der Geest LGM, et al. Effect of centralization and regionalization of pancreatic surgery on resection rates and survival. *Br J Surg*. Jul 23 2021;108(7):826-833. doi:10.1093/bjs/znaa146

8. Dutch Federation of Oncologic Societies (Stichting Oncologische Samenwerkin - SONCOS). Available via: <https://www.soncos.org/kwaliteit/normeringsrapport/>

9. National Guideline Pancreatic Cancer. Richtlijn Pancreas Carcinoom - Federatie Medisch Specialisten. Available at: <https://richtlijnendatabase.nl/richtlijn/pancreascarcinoom/startpagina.html>.

10. Tempero MA, Malafa MP, Al-Hawary M, et al. Pancreatic Adenocarcinoma, Version 2.2021, NCCN Clinical Practice Guidelines in Oncology. *Journal of the National Comprehensive Cancer Network*. 01 Apr. 2021 2021;19(4):439-457. doi:10.6004/jnccn.2021.0017

11. Clarke CA, Glaser SL, Leung R, Davidson-Allen K, Gomez SL, Keegan THM. Prevalence and characteristics of cancer patients receiving care from single vs. multiple institutions. *Cancer Epidemiology*. 2017/02/01/ 2017;46:27-33. doi:<https://doi.org/10.1016/j.canep.2016.11.001>

12. Shannon AB, Mo J, Song Y, et al. Does multicenter care impact the outcomes of surgical patients with gastrointestinal malignancies requiring complex multimodality therapy? *J Surg Oncol*. Jun 20 2020;doi:10.1002/jso.26075

13. Strijker M, Mackay TM, Bonsing BA, et al. Establishing and Coordinating a Nationwide Multidisciplinary Study Group: Lessons Learned by the Dutch Pancreatic Cancer Group. *Annals of Surgery*. 2020;271(4):e102-e104. doi:10.1097/sla.0000000000003779

14. Smits FJ, Verweij ME, Daamen LA, et al. Impact of Complications After Pancreatoduodenectomy on Mortality, Organ Failure, Hospital Stay, and Readmission: Analysis of a Nationwide Audit. *Ann Surg*. Jan 1 2022;275(1):e222-e228. doi:10.1097/sla.0000000000003835

15. Leyland A, Groenewegen P. *Multilevel Modelling for Public Health and Health Services Research*. Springer; 2020.

16. Neoptolemos JP, Moore MJ, Cox TF, et al. Effect of adjuvant chemotherapy with fluorouracil plus folinic acid or gemcitabine vs observation on survival in patients with resected periampullary adenocarcinoma: the ESPAC-3 periampullary cancer randomized trial. *Jama*. Jul 11 2012;308(2):147-56. doi:10.1001/jama.2012.7352

17. Valle JW, Palmer D, Jackson R, et al. Optimal duration and timing of adjuvant chemotherapy after definitive surgery for ductal adenocarcinoma of the pancreas: ongoing lessons from the ESPAC-3 study. *J Clin Oncol*. Feb 20 2014;32(6):504-12. doi:10.1200/jco.2013.50.7657

18. Gagliardi AR, Soong D, Gallinger S. Identifying Factors Influencing Pancreatic Cancer Management to Inform Quality Improvement Efforts and Future Research: A Scoping Systematic Review. *Pancreas*. Feb 2016;45(2):161-6. doi:10.1097/mpa.0000000000000484

19. Mackay TM, Smits FJ, Latenstein AEJ, et al. Impact of nationwide enhanced implementation of best practices in pancreatic cancer care (PACAP-1): a multicenter stepped-wedge cluster randomized controlled trial. *Trials*. Apr 16 2020;21(1):334. doi:10.1186/s13063-020-4180-z

20. Luijten J, Nieuwenhuijzen GAP, Sosef MN, et al. Impact of nationwide centralization of oesophageal, gastric, and pancreatic surgery on travel distance and experienced burden in the Netherlands. *Eur J Surg Oncol*. Feb 2022;48(2):348-355. doi:10.1016/j.ejso.2021.07.023

21. Stitzenberg KB, Sigurdson ER, Egleston BL, Starkey RB, Meropol NJ. Centralization of cancer surgery: implications for patient access to optimal care. *J Clin Oncol*. Oct 1 2009;27(28):4671-8. doi:10.1200/jco.2008.20.1715

22. Smith AK, Shara NM, Zeymo A, et al. Travel patterns of cancer surgery patients in a regionalized system. *J Surg Res*. Nov 2015;199(1):97-105. doi:10.1016/j.jss.2015.04.016

23. Symer MM, Abelson JS, Yeo HL. Barriers to Regionalized Surgical Care: Public Perspective Survey and Geospatial Analysis. *Ann Surg*. Jan 2019;269(1):73-78. doi:10.1097/sla.0000000000002556

24. den Engelsen B, Hatenboer D, Hoff JL. Influence of qualitystandards on accessibility of medical specalistic care. National Health Care Institute of The Netherlands (Zorginstituut Nederland), 19 February 2019.
